# Compounds Released by the Biocontrol Yeast *Hanseniaspora opuntiae* Protect Plants Against *Corynespora cassiicola* and *Botrytis cinerea*

**DOI:** 10.3389/fmicb.2018.01596

**Published:** 2018-07-17

**Authors:** Mariana Ferreira-Saab, Damien Formey, Martha Torres, Wendy Aragón, Emir A. Padilla, Alexandre Tromas, Christian Sohlenkamp, Kátia R. F. Schwan-Estrada, Mario Serrano

**Affiliations:** ^1^Centro de Ciencias Genómicas, Universidad Nacional Autónoma de Mexico, Cuernavaca, Mexico; ^2^Departemento de Agronomia, Universidade Estadual de Maringá, Maringá, Brazil

**Keywords:** Biocontrol agent, elicitors, *Hanseniaspora opuntiae*, *Corynespora cassiicola*, *Botrytis cinerea*, *Glycine max*, *Arabidopsis thaliana*, plant defense responses

## Abstract

Plant diseases induced by fungi are among the most important limiting factors during pre- and post-harvest food production. For decades, synthetic chemical fungicides have been used to control these diseases, however, increase on worldwide regulatory policies and the demand to reduce their application, have led to searching for new ecofriendly alternatives such as the biostimulants. The commercial application of yeasts as biocontrol agents, has shown low efficacy compared to synthetic fungicides, mostly due to the limited knowledge of the molecular mechanisms of yeast-induced responses. To date, only two genome-wide transcriptomic analyses have characterized the mode of action of biocontrols using the plant model *Arabidopsis thaliana*, missing, in our point of view, all its molecular and genomic potential. Here we describe that compounds released by the biocontrol yeast *Hanseniaspora opuntiae* (HoFs) can protect *Glycine max* and *Arabidopsis thaliana* plants against the broad host-range necrotrophic fungi *Corynespora cassiicola* and *Botrytis cinerea*. We show that HoFs have a long-lasting, dose-dependent local, and systemic effect against *Botrytis cinerea*. Additionally, we performed a genome-wide transcriptomic analysis to identify genes differentially expressed after application of HoFs in *Arabidopsis thaliana*. Our work provides novel and valuable information that can help researchers to improve HoFs efficacy in order for it to become an ecofriendly alternative to synthetic fungicides.

## Introduction

Of all food produced for human consumption, every year 1.3 billion tons are lost or wasted (http://www.fao.org). Only during post-harvest, 25 to 50% of the production can be lost due to plant diseases induced by microorganisms and by suboptimal handling and storage conditions (Nunes, 2012). Fungal species are responsible for most of these losses, including the genera *Alternaria, Aspergillus, Botrytis, Fusarium, Geotrichum, Gloeosporium, Penicillium, Mucor*, and *Rhizopus* (Barkai-Golan, 2001; Dean et al., 2012). The importance of fungi-related disease can be exemplified by mentioning that if producers could avoid the damages associated to fungi in the five most important crops, 600 million people could be fed each year (Fisher et al., 2012). For decades, fungicides have been used to control fungi-induced diseases. However, an increase in worldwide regulatory policies and the demand to reduce their application, due to potential harmful side effects to the environment and to humans, have led to searching for new ecofriendly alternatives. One of these alternatives is biostimulants, which are defined as a naturally-occurring chemicals or microorganisms that enhance plant development, abiotic, and biotic stress tolerance and/or crop quality traits (Du Jardin, 2015).

Biostimulants that protect the plant against pathogens can be classified as elicitors and biocontrol agents (BCAs). Microorganisms such as bacteria and yeast, have been used as BCAs to control herbivores and several plant pathogens. For instance, bacteria from the genera *Bacillus, Pseudomonas*, and *Pantoea* have been used to control mold-produced fungi, mainly by the production of antibiotics (Nunes et al., 2002; Cirvilleri et al., 2005; Ren et al., 2013). However, even if some of them are already used in the field, several concerns arrise, in particular the possible development of resistance in the pathogens. Nowadays, one alternative is to use yeast as BCAs, since they are antagonistic microorganisms that can grow under adverse environmental conditions without special nutrients requirements and do not produce compounds harmful to human health (Liu et al., 2013). The basis of the antagonistic properties of yeast against pathogens has been previously described and includes: competition for nutrients, pH changes on the plant surface, production of ethanol and biosynthesis of killer toxins called mycocins (Hatoum et al., 2012). Nevertheless, despite all these beneficial traits, the commercial application of yeast in the field as BCAs has shown an inconsistent efficacy compared to synthetic fungicides, mostly due to the lack of knowledge of the molecular mechanisms behind yeast-induced plant defense responses (Massart et al., 2015).

On the other hand, elicitors are chemical molecules that activate the plant defense responses, and include microbe- and damage-associated molecular patterns (MAMPs and DAMPs), polypeptides, glycoproteins, lipids, proteins, glycolipids, and oligosaccharides (Katagiri and Tsuda, 2010; Maffei et al., 2012; Hael-Conrad et al., 2015; Yin et al., 2016). Once the elicitors are perceived by the plant, the first line of defense, called plant innate immunity is activated. During this initial defense mechanism, the production of reactive oxygen species (ROS), calcium influx, MAPK-dependent signaling cascades, localized cell death and transcriptional induction of the early defense response genes are activated (Katagiri and Tsuda, 2010; Tsuda and Somssich, 2015). After the induction of innate immunity at the local infected tissue, secondary defense responses are triggered, including salicylic the acid- (SA), jasmonic acid- (JA), and ethylene- (ET) dependent signaling pathways, that lead to the activation of systemic acquired resistance (SAR) at non-infected distal parts of the plant (Boller and Felix, 2009; Robert-Seilaniantz et al., 2011). The combined effect of the local and systemic defense responses, can block efficiently the disease inflicted by non-adapted pathogens (Craig et al., 2009). Due to these characteristics, elicitors have the potential to be used in agriculture as alternative to fungicides. However, to do so, it is necessary to better characterize the molecular changes induced by elicitors in order to optimize its application and activity in the field (Wiesel et al., 2014).

Molecular characterization of the plant-microbe interactions has been greatly benefitted from the technical advances in areas including metabolomics, proteomics, genomics and bioinformatics, in particular using *Arabidopsis thaliana* as a model. For example, this has led to novel conceptual advances in the understanding of the molecular basis of plant-pathogen interactions (Mishra et al., 2017). Importantly, these advances also saw the dawn of a series of potential applications that could impact crop protection (Bhadauria, 2016). During the last decade, several genome-wide transcriptomic analyses have been used to characterize the mode of action of BCAs (Massart et al., 2015). However, strangely, many of these analyses have been performed under *in-vitro* conditions and only two of them were characterized using the interaction *Arabidopsis thaliana*-BCAs as pathosystem (Feng et al., 2012; Morán-Diez et al., 2012).

### HoFs

In this report, we show that compounds released by the biocontrol yeast *Hanseniaspora opuntiae*, henceforth identified as *H. opuntiae*-Filtrates (HoFs), have the potential to protect against the broad host-range necrotrophic fungi *Corynespora cassiicola* and *Botrytis cinerea*. In order to better understand the molecular basis of HoFs-induced resistance, we characterized its activity in the well-described pathosystem *Arabidopsis thaliana-Botrytis cinerea*. We determined that HoFs can protect *Arabidopsis thaliana* against the necrotrophic fungus *Botrytis cinerea*. HoFs can induce the defense response in a dose-dependent manner. Additionally, performing a genome-wide transcriptomic analysis (RNA-seq), we identified that the genes differentially expressed upon application of HoFs, differ from those induced by other previously-described BCAs. This valuable information might help to reveal the molecular mechanisms behind HoFs-induced defense and can help researchers to improve their efficacy and to become an ecofriendly alternative to pesticides.

## Materials and methods

### Purification of HoFs

*Hanseniaspora opuntiae* CCMA 0760, was provided by the laboratory of Physiology and Genetics of the Federal University of Lavras, Brazil. *Hanseniaspora opuntiae* was grown in YNB (Yeast Nitrogen Base) media for 10 days in a 12 h light/12 h dark cycle at 24°C. At the end of the growth period, the culture media was centrifuged at 10,000 rpm for 20 min and the supernatant was filtered using 0.22 μm filters. Filtered material (HoFs) was diluted at the indicated concentration with distilled sterile water. In order to have a weight/volume concentration, the filtrated material (100%) was lyophilized and the concentration was determined (8.45 mg/ml).

### *In-vitro* inhibitory assay of *Corynespora cassiicola* and *Botrytis cinerea* growth

*Corynespora cassiicola* growth and preparation of spore suspension were performed as previously described (Soares et al., 2009). *Botrytis cinerea* strain BMM was provided by Brigitte Mauch-Mani (University of Neuchatel, Switzerland). *Botrytis cinerea* growth and preparation of spore suspension were performed as previously described (L'Haridon et al., 2011). For the inhibitory assay, a spore suspension of *Corynespora cassiicola* (3 × 10^5^ spores ml^−1^) or *Botrytis cinerea* (5 × 10^4^ spores ml^−1^) was placed at the center of a Petri dish containing potato dextrose agar media (PDA) supplemented with 20, 30, 40, and 50% HoFs and incubated at 22°C for 72 h. Inhibition was evaluated by measuring the diameter of the mycelium on the dish. The experiment was carried out in a completely randomized design (CRD), with five replicates for each treatment. *Botrytis cinerea* spore germination assay was performed as previously described (Hael-Conrad et al., 2015). Pictures were taken at 24 hpi with a digital camera attached to a Leica DMR microscope with bright-field settings. Images of growing *Botrytis cinerea* hyphae were analyzed using Image J version 1.51 (NIH).

### Plant maintenance

*Glycine max* plants cultivar INT 6100, were grown under greenhouse conditions on pots containing non-autoclaved soil. *Arabidopsis thaliana* seeds were grown on a pasteurized soil mix of humus and perlite (3:1), kept at 4°C for 2 days and then transferred to the growth chamber. Plants were grown during 4 weeks in a 12 h light/12 h dark cycle with 60–70% of relative humidity, at a day temperature of 20–22°C and a night temperature of 16–18°C. *Arabidopsis thaliana* ecotype Columbia-0 (Col-0) was obtained from the Nottingham Arabidopsis Stock Centre (Nottingham, UK).

### HoFs treatment and *Corynespora cassiicola* or *Botrytis cinerea* plant inoculation

*Corynespora cassiicola* infection procedure and disease severity quantification were performed as previously described (Soares et al., 2009). *Glycine max* plants were grown until the V4 developmental stage (third fully expanded trifolium) and sprayed until saturation with 20% HoFs or mock (distilled sterile water) every 7 days, for 4 weeks. 24 h after the last treatment, plants were infected with a *Corynespora cassiicola* spore suspension (3 × 10^5^ spores ml^−1^) and 120 h post infection (hpi) disease severity was measured determining the minimum and maximum limits and the intermediate levels of the scale, according to Weber-Fechner's stimulus-response law, as previously described (Soares et al., 2009). *Botrytis cinerea* infection procedure and lesion size measurement were performed as previously described (L'Haridon et al., 2011). Four-week-old *Arabidopsis thaliana* plants were sprayed until saturation with 50% HoFs or mock (YNB) for 24, 48, 72, 96, or 120 h post treatment (hpt), as indicated in the Figure legends. After this time, 3 μl droplets containing *Botrytis cinerea* spore suspension (5 × 10^4^ spores ml^−1^) were applied. Infection symptoms were evaluated 72 hpi by measuring lesion size (cm). For the dose-response assay, plants were pre-treated with the indicated concentration HoFs and evaluated at 72 hpi. For the systemic assay, plants were pre-treated (watering the soil until saturation) with 50% HoFs or mock, and 24 hpt leaves were infected with *Botrytis cinerea* and evaluated at 72 hpi.

### RNA extraction

*Arabidopsis thaliana* leaves from 5 plants were harvested 24 hpt, pooled and immediately frozen in liquid nitrogen and kept at −80°C until use. Total RNA was extracted using the Spectrum^TM^ Plant total RNA Kit (www.sigmaaldrich.com) as described in the manufacturer's protocols. The integrity of extracted RNA was measured by agarose gel electrophoresis (1.2%), concentrations and purity were determined by NanoDrop 2000/2000c (Thermo Fisher Scientific). Samples used for RNA-seq were also analyzed using an Agilent 2100 Bioanalyzer (Agilent Genomics).

### Genome-wide transcriptomic analysis

The RNA-seq libraries were prepared from isolated total RNA from 5 plants, pooled from three independent experiments, using the Illumina TruSeq RNA Sample Preparation Kit (Illumina, San Diego, CA, USA) following the manufacturer's instructions. The libraries were sequenced using an Illumina GAIIx platform for 72 paired-end cycles following the manufacturer's protocol. Sequences are publicly available through the Gene Expression Omnibus database under the accession number GSE113810 (https://www.ncbi.nlm.nih.gov/geo/query/acc.cgi?acc=gse113810). Contamination and adapter removal was carried out using in-house Perl scripts. Fastq sequences were filtered based on quality (FASTQ Quality Filter v0.0.6, Q 33, http://hannonlab.cshl.edu/fastx_toolkit/index.html) and mapped on *Arabidopsis thaliana* transcriptome (TAIR10) using Bowtie2 (Langmead and Salzberg, 2012). Gene expression was calculated using RSEM v1.3 (Li and Dewey, 2011) and compared between the two RNA-seq libraries using DEGseq v3.6 (Wang et al., 2010), and the FPKM data from RSEM. Only transcripts with a Log2 fold change < −1 or > 1 with a *p*-value < 0.05 were considered. DEGs identified in by genome-wide transcriptomic analysis were analyzed and classified into gene ontology classes (GO) using the analysis toolkit agriGO (http://bioinfo.cau.edu.cn/agriGO/) previously described (Du et al., 2010). Identification of commonly regulated DEGs from previously published data and from the present work were performed using the software FiRe ver. 2.2 as previously described (Garcion and Metraux, 2006).

### Real time RT-PCR

Pooled total RNA (1.0 μg) from 5 plants, from two independent experiments, was retro-transcribed into cDNA according to the manufacturer's indications using the SCRIPT cDNA Synthesis Kit (Jena Bioscience www.jenabioscience.com). RT-qPCR was performed in 96-well plates with the Applied Biosystems StepOne™ and StepOnePlus™ Real-Time PCR System (ThermoFisher Scientific), using SYBR Green Maxima SYBR Green/ROX qPCR Master Mix (2X) (ThermoFisher Scientific, www.thermofisher.com). Two independent experiments were analyzed with three technical replicates each. RT-qPCR conditions were as follows: an initial 95°C denaturation step for 15 min followed by denaturation for 15 s at 95°C, annealing for 30 s at 60°C, and extension for 30 s at 72°C for 45 cycles. Gene expression values were normalized using the mean expression of two genes: AT4G26410 and AT1G72150 previously described as stable reference genes (Serrano and Guzmán, 2004; Czechowski et al., 2005). Normalized gene expression was determined using the comparative 2^−ΔΔ*CT*^ method previously described (Schmittgen and Livak, 2008). Primers for *ACS6, PR4*, and *PDF1.2* gene expression were previously described (Hael-Conrad et al., 2015).

## Results

### Compounds released by *Hanseniaspora opuntiae* protect against the plant pathogen *Corynespora cassiicola*

**Figure 1:**
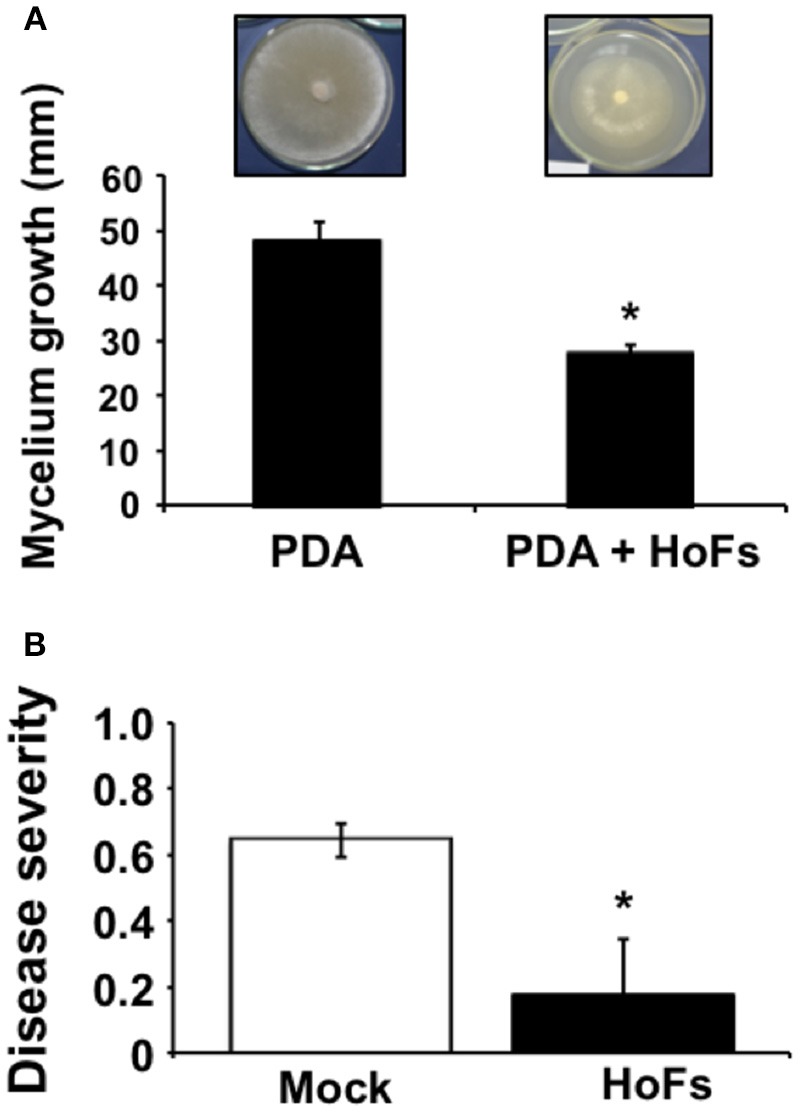
HoFs inhibit *Corynespora cassiicola* growth *in vitro* and protect *Glycine max* plants against this pathogen. **(A)** Spore suspension of *Corynespora cassiicola* (3 × 10^5^ spores ml^−1^) was placed on the center of the Petri dish containing PDA (Mock) or PDA supplemented with 20% HoFs and incubated at 22°C for 72 h. Growth inhibition was evaluated measuring the diameter of the mycelium on the dish. Representative pictures of the inhibitory assay are included above each histogram as a visual illustration. **(B)** Soybean plants were grown until the V4 developmental stage; afterwards, sprayed until saturation with distilled sterile water (Mock) or 20% HoFs every 7 days, for 4 weeks. 24 h after the last treatment, plants were infected with *Corynespora cassiicola* spore suspension (3 × 10^5^ spores ml^−1^) and disease severity was determined 120 hpi, as previously described (Soares et al., 2009). Bars represent mean values (± SD) of three independent experiments. Asterisks indicate a statistically significant difference between Mock- and HoFs-treated sample*s*, according to the Student's *T*-test (*P* ≤ 0.05).

Yeasts have been characterized as biocontrol agents (BCAs) and eco-friendly alternatives to commercial pesticides against different plant pathogens (Liu et al., 2013); in particular, the antimicrobial compounds released, known as antifungal killer toxins or “mycocins” (Hatoum et al., 2012). In order to identify potential BCAs, a collection of yeast resident on *Theobroma cacao* fruits was isolated and the antimicrobial compounds released were tested against the fungal plant pathogen *Corynespora cassiicola* (Ferreira-Saab, 2018). One of the potential BCAs identified was *Hanseniaspora opuntiae*, which has been previously identified as part of the microbiome present in the cocoa bean fermentation process (Papalexandratou et al., 2013). In order to study the potential of *Hanseniaspora opuntiae* as biocontrol agent, *Corynespora cassiicola* spores were germinated on PDA media supplemented with 20% of compounds released by this yeast, identified as HoFs. *In-vitro* mycelia growth was inhibited by approximately 50%, compared to the PDA control media (Figure 1A). *Corynespora cassiicola* has been described as an important pathogen of many crop plants, including soybean (*Glycine max*). Then we determined if HoFs extended their biocontrol effect on this crop. Soybean plants were treated with 20% HoFs and after 24 hpt, infected with *Corynespora cassiicola* and at 120 hpi disease severity was quantified as previously described (Soares et al., 2009). A reduction of approximately 75% in disease severity, compared to the mock-treated control plants, was induced by HoFs 120 hpi (Figure 1B). These results indicated that HoFs not only inhibited *Corynespora cassiicola* growth *in-vitro*, but can be also used as BCAs on soybean plants.

### The pathosystem *Arabidopsis thaliana-botrytis cinerea* can be used as a model to analyze the HoFs-induced defense mechanisms

**Figure 2:**
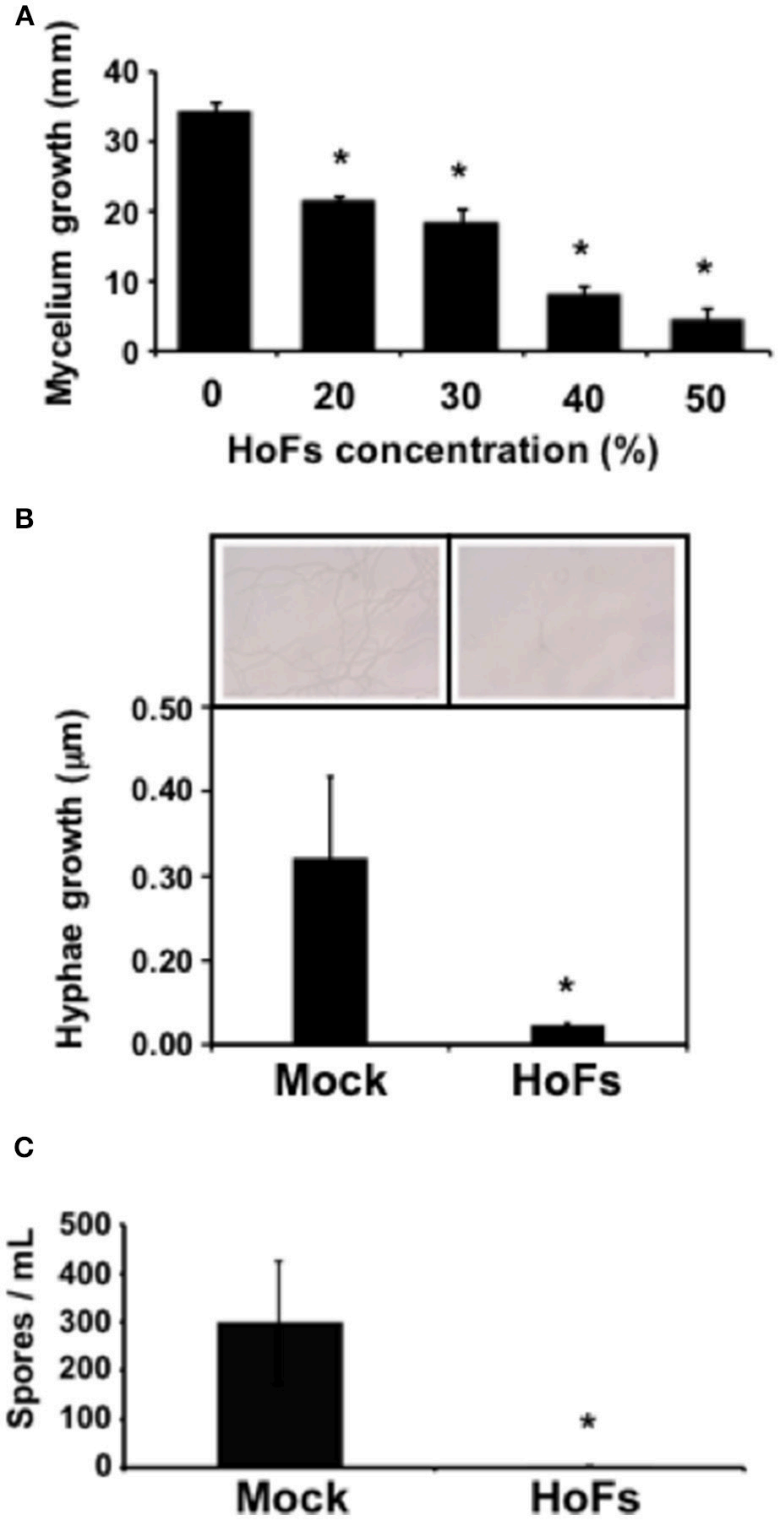
HoFs induced a *Botrytis cinerea* development inhibition. **(A)** Spore suspension of *Botrytis cinerea* (5 × 10^4^ spores ml^−1^) was placed on the center of the Petri dish containing PDA supplemented with indicated concentrations of HoFs and incubated at 22°C. Growth inhibition was evaluated measuring the diameter of the mycelium on the dish 72 hpi. **(B)** Hyphae elongation produced by *Botrytis cinerea*, grown on 20% HoFs 24 hpi, was quantified as previously described (Hael-Conrad et al., 2015). A representative image of each treatment is presented. **(C)** Spores produced by *Botrytis cinerea* 15 days after the grown on 20% HoFs, were isolated and quantified as previously described (L'Haridon et al., 2011). Bars represent mean values (± SD) of three independent experiments. Asterisks indicate a statistically significant difference between 0% and the indicated concentrations of HoFs, according to the Student's *T*-test (*P* ≤ 0.05).

**Figure 3:**
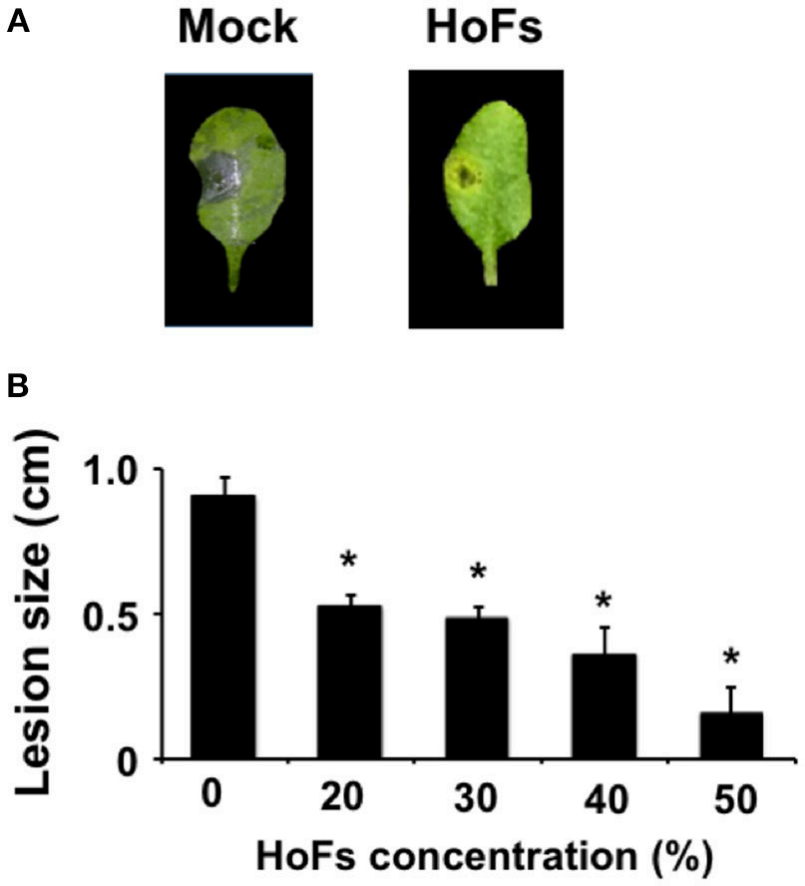
HoFs protect *Arabidopsis thaliana* plants against *Botrytis cinerea*. **(A)** 4-week-old *Arabidopsis thaliana* plants were sprayed until saturation with YNB medium (Mock) or 50% HoFs. Twenty-Four hpt 3 μl droplets containing a *Botrytis cinerea* spore suspension (5 × 10^4^ spores ml^−1^) were applied and infection symptoms were evaluated 72 hpi. Representative pictures of the inhibitory assay are included as a visual illustration. **(B)** Four-week-old *Arabidopsis thaliana* plants were treated with the indicated HoFs concentration and infected as indicated above. Infection symptoms were evaluated 72 hpi by measuring lesion size (cm). Bars represent mean values (± SD) of three independent experiments each with twenty replicates. Asterisks indicate a statistically significant difference between Mock- and HoFs-treated sample*s*, according to the Student's *T*-test (*P* ≤ 0.05).

**Figure 4:**
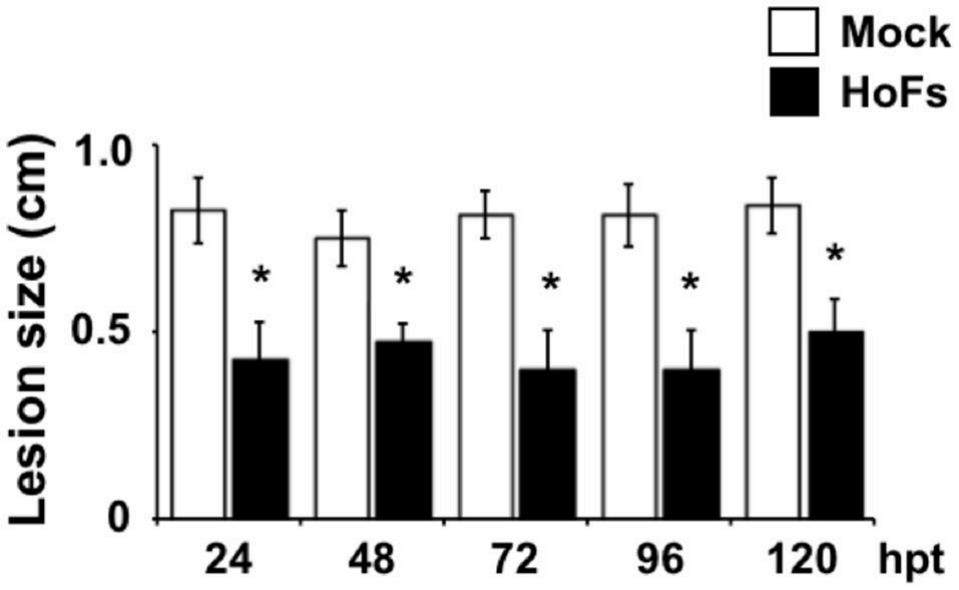
Time-course growth inhibition of *Botrytis cinerea* in *Arabidopsis thaliana* plants treated with HoFs. 4-week-old *Arabidopsis thaliana* plants were sprayed until saturation with YNB medium (Mock) or 50% HoFs for 24, 48, 72, 96, and 120 (hpt), after these times 3 μl droplets containing *Botrytis cinerea* spore suspension (5 × 10^4^ spores ml^−1^) were applied. Infection symptoms were evaluated 72 hpi by measuring lesion size (cm). Bars represent mean values (± SD) of three independent experiments each with twenty replicates. Asterisks indicate a statistically significant difference between Mock- and HoFs-treated sample*s*, according to the Student's *T*-test (*P* ≤ 0.05).

In the field, application of BCAs has shown an inconsistent efficacy compared with synthetic chemical compounds and one possibility, to avoid this problem, is to better understand the molecular mechanisms behind the application of BCAs (Massart et al., 2015). In order to characterize the molecular mechanisms underlying the HoFs-induced biocontrol effect, we used the well-characterized plant-pathosystem *Arabidopsis thaliana*–*Botrytis cinerea*. First, we determined if HoFs inhibited the development of the necrotroph pathogen under *in-vitro* conditions (Figure 2). Analyzing a dose-dependent response, we observed that *Botrytis cinerea* grown on PDA media supplemented with 20 and 30% HoFs, showed about 25% inhibition of mycelial growth (Figure 2A). Increasing HoFs concentration up to 40 and 50%, directly correlated with a higher reduction of mycelial growth (between 70 and 80% inhibition, respectively), showing a dose-dependent response induced by HoFs (Figure 2A). To determine if HoFs directly affect the germination and the production of *Botrytis cinerea spores*, we analyzed the development of the fungus in the presence of 20% HoFs (Figures 2B,C). We determined that spores can germinate at 20% HoFs, but hyphae growth was inhibited (Figure 2B). Additionally, we observed that mycelia developed under this conditions did not further produce spores (Figure 2C). These results suggest that HoFs have antifungal effect on *Botrytis cinerea*. Next, 4-week-old *Arabidopsis thaliana* plants were pre-treated with 50% HoFs 24 hpt and then infected with *Botrytis cinerea*. We observed a strong inhibition of the lesion caused by this pathogen on HoFs-treated plants compared to mock-treated samples, 72 hpi (Figure 3A). Additionally, a similar dose-dependent effect, observed under *in-vitro* conditions (Figure 2A), was determined *in planta*, since at higher HoFs concentration a smaller lesion size was quantified (Figure 3B). Then, to evaluate for how long HoFs can protect *Arabidopsis thaliana* plants against *Botrytis cinerea*, different hpt were assayed, measuring the lesion size at 72 hpi. For all of the times analyzed (24 to 120 hpt), HoFs-treated plants showed significant differences compared to mock-treated control samples (Figure 4), indicating that HoFs induced a protective effect over the plant-pathogen interaction at all of these time points. Taken together, these results indicated that HoFs protect *Arabidopsis thaliana* against *Botrytis cinerea* and that this pathosystem can be used as a model to characterize the molecular changes induced by HoFs application.

### HoFs induced a systemic protection against *Botrytis cinerea*

**Figure 5:**
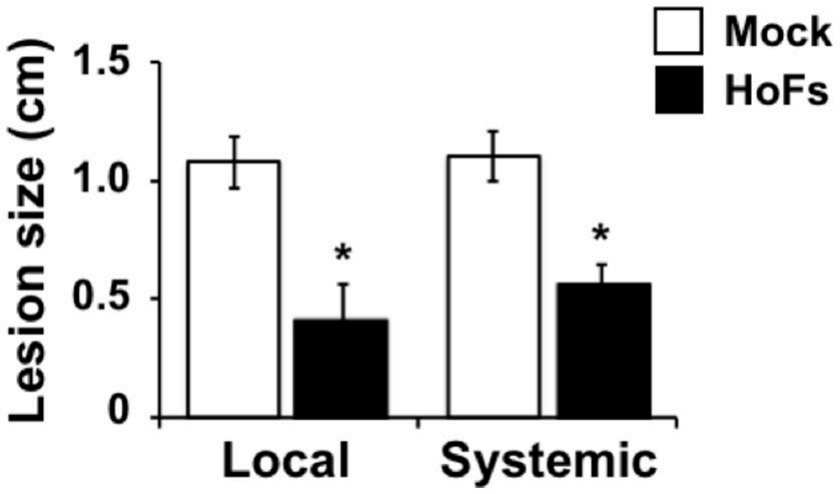
Systemic effect induced by HoFs against *Botrytis cinerea* in *Arabidopsis thaliana* plants. 4 week-old *Arabidopsis thaliana* plants were pre-treated in the roots (watered soil until saturation) with YNB medium (Mock) or 50% HoFs and 24 hpt leaves infected with *Botrytis cinerea* and evaluated at 72 hpi. Bars represent mean values (± SD) of three independent experiments each with twenty replicates. Asterisks indicate a statistically significant difference between Mock- and HoFs-treated sample*s*, according to the Student's *T*-test (*P* ≤ 0.05).

Under *in-vitro* conditions we observed an antifungal effect on *Botrytis cinerea* growth (Figure 2), this observation rises the questions of whether the protective effect observed *in planta* was induced by the direct effect of HoFs localized on the local leaf surface or by the modification of the plant defense responses itself. In order to clarify this question, we applied HoFs directly to the roots and we infected the untreated leaves (systemic) with *Botrytis cinerea*. 72 hpi HoFs-root-treated plants showed a similar significant reduction of lesion size, as the local HoFs-treated leaves (Figure 5). These results suggest two possibilities: (1) HoFs can be transported from the roots to the the entire plant, inhibiting *Botrytis cinerea* due to their antifungal effect and (2) HoFs might play a role as a potential elicitor of the defense responses that leads to a systemic resistance against the necrotrophic pathogen *Botrytis cinerea*. Either way, these result indicated that application of HoFs can triggered a systemic protection against this pathogen.

### HoFs induced a reprograming of the *Arabidopsis thaliana* transcriptome

**Figure 6:**
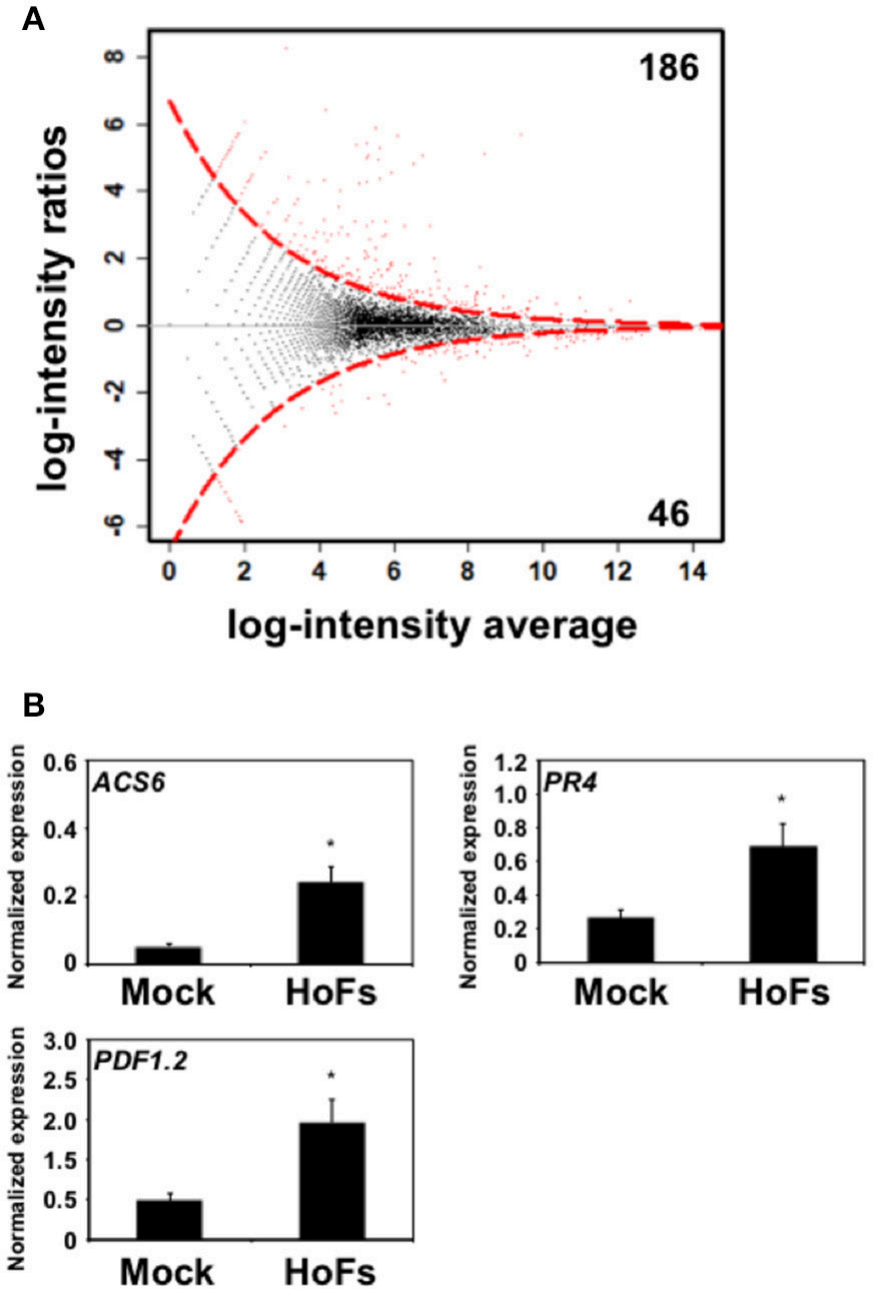
Genome-wide transcriptomic analysis of HoFs-induced *Arabidopsis thaliana* plants. Five *Arabidopsis thaliana* 4-week-old plants, were sprayed until saturation with YNB medium (Mock) or 50% HoFs and total RNA, from three independent experiments, were pooled and sequenced (RNA-seq). **(A)** MA-plot of Mock- vs. HoFs-treated samples. The red points are the genes identified as differentially expressed (*p*-value < 0.05). Black dots represent genes with similar expression. The discontinued red line represents the limit between similarly and differentially expressed genes. The black horizontal line at zero provides a visual check for symmetry. **(B)** Quantitative real-time PCR (RT-qPCR) analysis of JA- and ET-induced genes. Expression of *ACS6, PR4* and *PDF1.2* was determined and normalized with respect to the mean of two reference genes AT4G26410 and AT1G72150, as previously described (Serrano and Guzmán, 2004; Hael-Conrad et al., 2015). The value in each histogram is the mean (± SE) of two independent experiments (*n* = 10) with three technical replicates for each RT-qPCR assay. Asterisks indicate a statistically significant differences between Mock- and HoFs-treated sample*s*, according to Student's *T*-test (*p* < 0.05).

During the last decade large-scale transcriptomic analysis have been used to understand how BCAs improve plant health (Massart et al., 2015). However, to our knowledge, only few a studies have used *Arabidopsis thaliana* as a model (Feng et al., 2012; Morán-Diez et al., 2012). In order to discover the transcriptional modifications induced by HoFs, the transcriptome of HoFs-treated plants was analyzed by RNA-seq (Supplementary Table 1, Figure 6). The expression of 186 and 46 genes was down- or up-regulated, respectively in HoFs-treated plants compared to non-induced samples (Figure 6A). GO analysis revealed that the most significant differentially expressed genes (DEGs), induced and repressed belonged to response to stress, chemical and abiotic stimulus, among others (Table 1).

**Table 1:** Gene ontology (GO) enrichment analysis of differentially expressed genes of HoFs-treated *Arabidopsis thaliana* plants.

**GO ID**	**Description**	**No. Genes**	***p*-Value**
**(A)**			
GO:0009628	Response to abiotic stimulus	9	5.90E-06
GO:0050896	Response to stimulus	14	7.10E-06
GO:0006950	Response to stress	11	5.00E-06
GO:0042221	Response to chemical stimulus	10	1.40E-05
GO:0006810	Transport	7	1.30E-03
GO:0051234	Establishment of localization	7	1.40E-03
GO:0051179	Localization	7	1.70E-03
GO:0009725	Response to hormone stimulus	5	2.00E-03
GO:0009719	Response to endogenous stimulus	5	2.90E-03
GO:0010033	Response to organic substance	5	7.60E-03
GO:0022891	Substrate-specific transmembrane transporter activity	5	1.40E-03
GO:0022892	Substrate-specific transporter activity	5	2.80E-03
GO:0022857	Transmembrane transporter activity	5	3.70E-03
GO:0005215	Transporter activity	5	1.10E-02
**(B)**			
GO:0006950	Response to stress	51	1.10E-23
GO:0050896	Response to stimulus	61	2.50E-20
GO:0015979	Photosynthesis	16	3.80E-17
GO:0042221	Response to chemical stimulus	41	3.60E-17
GO:0006091	Generation of precursor metabolites and energy	18	5.90E-16
GO:0009611	Response to wounding	14	2.60E-13
GO:0010033	Response to organic substance	29	3.30E-13
GO:0009605	Response to external stimulus	18	4.70E-13
GO:0044237	Cellular metabolic process	77	5.90E-13
GO:0019684	Photosynthesis, light reaction	11	1.70E-12
GO:0010200	Response to chitin	12	4.00E-12
GO:0009987	Cellular process	89	9.70E-12
GO:0008152	Metabolic process	83	2.80E-11
GO:0009409	Response to cold	14	1.70E-10
GO:0009743	Response to carbohydrate stimulus	12	6.40E-10
GO:0009607	Response to biotic stimulus	17	1.90E-09
GO:0009266	Response to temperature stimulus	15	2.60E-09
GO:0044249	Cellular biosynthetic process	48	5.60E-09
GO:0031408	Oxylipin biosynthetic process	6	1.00E-08
GO:0009628	Response to abiotic stimulus	24	1.10E-08
GO:0009145	Purine nucleoside triphosphate biosynthetic process	7	1.50E-08
GO:0009142	Nucleoside triphosphate biosynthetic process	7	1.60E-08
GO:0009144	Purine nucleoside triphosphate metabolic process	7	1.60E-08
GO:0009141	Nucleoside triphosphate metabolic process	7	2.00E-08
GO:0009058	Biosynthetic process	48	1.90E-08
GO:0031407	Oxylipin metabolic process	6	3.00E-08
GO:0009620	Response to fungus	9	3.30E-08
GO:0051707	Response to other organism	15	3.90E-08
GO:0009414	Response to water deprivation	10	6.00E-08
GO:0009150	Purine ribonucleotide metabolic process	7	7.40E-08
GO:0009415	Response to water	10	9.20E-08
GO:0006164	Purine nucleotide biosynthetic process	7	1.60E-07
GO:0006952	Defense response	16	1.50E-07
GO:0006163	Purine nucleotide metabolic process	7	1.80E-07
GO:0006970	Response to osmotic stress	12	1.80E-07
GO:0009259	Ribonucleotide metabolic process	7	2.40E-07
GO:0009695	Jasmonic acid biosynthetic process	5	2.70E-07
GO:0015992	Proton transport	6	2.90E-07
GO:0006818	Hydrogen transport	6	2.90E-07

*Biological process identified to be **(A)** down-regulated and **(B)** up-regulated on HoFs-treated compared to Mock-treated samples*.

GO analysis revealed that induced DEGs belonged to a group responsive to chitin, defense response, response to fungus and jasmonic acid (JA) biosynthetic processes (Table 1B). Previous reports have shown that *Arabidopsis thaliana* defense responses to *Botrytis cinerea* are JA- and ET-dependent (Thomma et al., 2001; Ferrari et al., 2003; Glazebrook, 2005). In order to validate the transcriptomic analysis, we compared the expression of JA- and ET-related genes, that were induced by HoFs (Figure 6B). Gene expression of the enzyme catalyzing the first and rate-limiting step of ET biosynthesis, 1-*aminocyclopropane-1-carboxylate synthase 6* (*ACS6*), the ET-responsive gene *PATHOGENESIS-RELATED 4* (*PR4*) and ET- and JA-responsive plant defensin gene (*PDF1.2*) were measured (Figure 6B). *ACS6, PR4* and *PDF1.2* have been previously described to be expressed during *Botrytis cinerea* infection (Windram et al., 2012; Hael-Conrad et al., 2015) and in agreement with these observations, we detected an up-regulation of these genes in HoFs-treated plants, compared to mock-treated samples (Figure 6B). These results help us to validate our genome-wide analysis and indicated that resistance to *Botrytis cinerea* induced by HoFs application, can be mediated, at least partially, by the transcriptional reprograming of the plant defense responses, in particular JA- and ET-induced pathway. This valuable information (Supplementary Table 1) can be used to uncover the HoFs-induced defense responses.

### Transcriptional reprograming induced by HoFs is different than other BCAs previously reported

**Figure 7:**
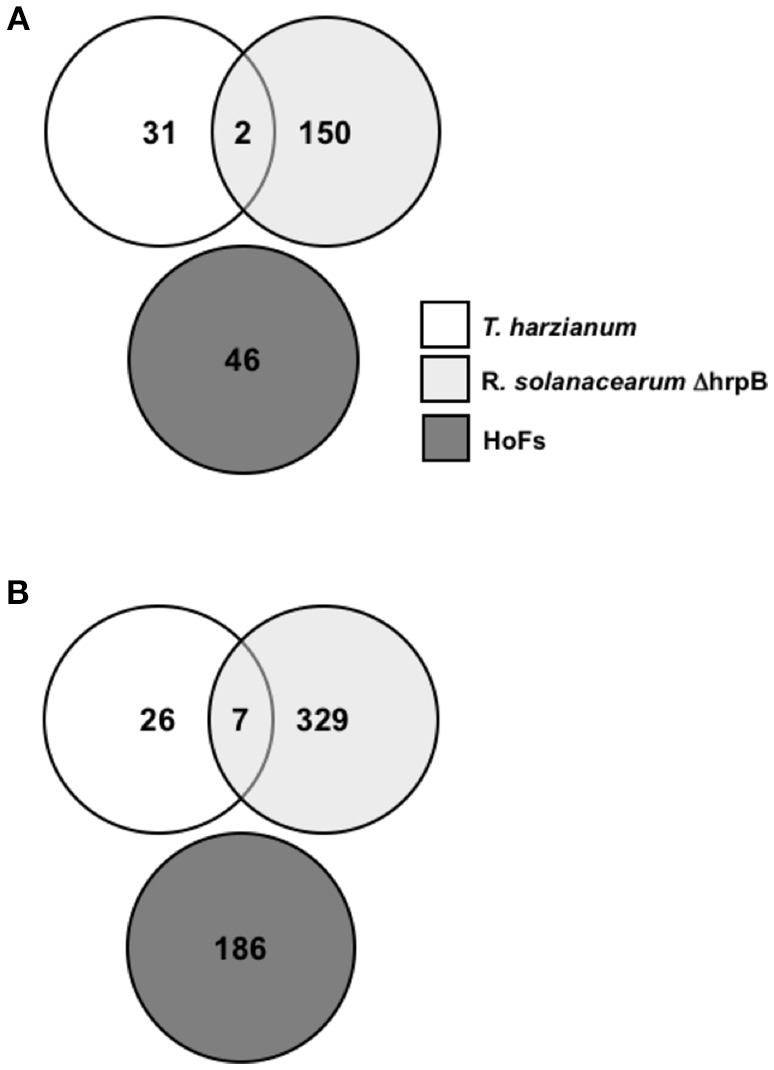
HoFs-induced differentially expressed genes (DEGs) have not been previously identified as part of BCAs-induced defense mechanisms. **(A)** and **(B)** Venn diagrams representing overlapping or non-overlapping gene sets of differentially down- and up-regulated genes respectively, previously identified in *Arabidopsis thaliana* plants induced for 24 hpi with *Trichoderma harzianum* (Morán-Diez et al., 2012)*, Ralstonia solanacearum* ΔhrpB mutant strain (Feng et al., 2012) or HoFs, as indicated.

Two genome-wide transcriptomic analysis have been performed to characterize the mode of action of BCAs using *Arabidopsis thaliana* as a model. The first, analyzed the transcriptome changes induced by the pre-inoculation (24 hpi) of *Arabidopsis thaliana* plants with *Ralstonia solanacearum* ΔhrpB mutant strain, which has been previously shown to protect against the virulent strain of this phytopathogenic root bacteria on tomato (Frey et al., 1994). From this analysis 152 and 336 genes were identified to be down- and up-regulated, respectively (Feng et al., 2012). Interestingly, 26% of the up-regulated genes were related to biosynthesis of abscisic acid (ABA) and signaling, suggesting an important role of this plant hormone on the defense mechanisms induced by this BCA (Feng et al., 2012). The other, described the transcriptomic response of *Arabidopsis thaliana* plants after inoculation with the biocontrol fungus *Trichoderma harzianum* at 24 hpi (Morán-Diez et al., 2012). From this analysis, only 66 DEGs were identified, 33 up- and 33 down-regulated as a result of the interaction (Morán-Diez et al., 2012). The expression of SA- and JA-related genes was down regulated, while genes involved in the abiotic stresses were induced (Morán-Diez et al., 2012). Here, in order to identify if treatments with BCAs share a similar transcriptomic signature, we analyzed the commonly co-expressed DEGs in *Arabidopsis thaliana* plants treated for 24 hpi with *Ralstonia solanacearum* ΔhrpB mutant strain, 24 hpi with *Trichoderma harzianum* and 24 hpt with HoFs (Figure 7). Only 2 and 7 genes were down- and up-regulated, respectively, after the pre-inoculation with ΔhrpB mutant and infection with *Trichoderma harzianum* (Figure 7), indicating that the two biocontrols triggered different defense response pathways. However, is worth to mentioning that we determined that DEGs induced or repressed by HoFs are not part of the same core of genes regulated by these other BCAs (Figure 7). These results indicate that HoFs-induced DEGs have not been previously identified as part of BCAs-induced defense mechanisms.

## Discussion

### HoFs have the potential to protect against the broad host-range necrotrophic fungi *Corynespora cassiicola* and *Botrytis cinerea*

The necrotrophic fungi *Corynespora cassiicola* and *Botrytis cinerea* are considered as important plant pathogens that affect pre- and post-harvest processes. *Corynespora cassiicola* resides on plant surfaces, nematodes cysts and human skin and can infect at least 530 plant species, including several important crops such as cowpea, cucumber, papaya, rubber, soybean and tomato (Dixon et al., 2009). While *Botrytis cinerea*, is a broad host-range necrotrophic fungus, commonly known as gray mold, that can infect more than 200 plant species, and for this, it has been classified as the second most important phytopathogen (Dean et al., 2012). Several elicitors have been previously described to protect the plants against *Botrytis cinerea*, including rhamnolipids, oligogalacturonides, chitosan, ceratoplatanin and the proteins PebC1 and AsES (Trotel-Aziz et al., 2006; Ferrari et al., 2007; Sanchez et al., 2012; Baccelli et al., 2014; Zhang et al., 2014; Feng et al., 2015; Hael-Conrad et al., 2015). However, to our knowledge, there is only one report where biocontrol agents were analyzed for their effect against *Corynespora cassiicola* under *in-vitro* and in field conditions. This early study, included the microorganisms *Trichoderma spp*., *Bacillus subtilis*, and *Pseudomonas florescence* and the elicitors from garlic bulb and neem seed kernel extracts (Manju et al., 2014). In our work, we determined that the elicitors released by the biocontrol yeast *Hanseniaspora opuntiae* (HoFs) can protect *Glycine max* and *Arabidopsis thaliana* plants against the necrotroph pathogens *Corynespora cassiicola* and *Botrytis cinerea*, respectively. Under *in-vitro* and *in-planta* conditions, HoFs show a dose-dependent behavior, similar to other elicitors previously characterized (Trotel-Aziz et al., 2006; Hael-Conrad et al., 2015). Additionally, we determined that the HoFs-induced protective effect on *Arabidopsis thaliana* plants against *Botrytis cinerea*, can be induced after 24 h pretreatment and maintained without significant reduction for up to 5 days (Figure 4). Taken together, these results indicated that HoFs have the potential to be used as biocontrols against these agronomically important pathogens. Furthermore, it will be interesting to study if HoFs can protect against other pathogens, including other fungi, bacteria and/or herbivores.

### HoFs induce local and systemic protection against *Botrytis cinerea*

HoFs show a protective effect *in-planta*, but additionally, they also inhibited the development of the pathogens under *in-vitro* conditions (Figures 1, 2). These results suggest that HoFs might work as fungicides, however, since we also observed a systemic protection in *Arabidopsis thaliana* plants (Figure 5), we can not discard the idea that HoFs can either be diffused through the whole plant and/or that, once inside the plant cell, they can induce the defense responses as true elicitors. The possibility that HoFs might act as elicitors inducing the defense responses is supported by the changes in the genome-wide transcriptomic machinery, since genes of the JA- and ET-related pathways that have been previously reported to be involved in the *Botrytis cinerea* response, are induced (Figure 6). Interestingly, a similar *in-vitro* inhibitory effect on *Botrytis cinerea* and the induction of the defense responses have been observed with other well-characterized elicitor, the chitosan (Trotel-Aziz et al., 2006). Exogenous application of elicitors has diverse and, sometimes, contradictory effect. While chitosan has been described to improve plant growth (Yin et al., 2016), constitutive activation of the defense responses by oligogalacturonides (OGs), have been recently shown to affect the plant growth rate, suggesting a defense-growth trade-off (Benedetti et al., 2018). Now, the question if HoFs have a similar effect is still open. Either way, the local and systemic protection induced by HoFs, might facilitate their application and might give them the potential to be used on the field to protect the crops against these pathogens.

### HoFs might induce systemic protection against *Botrytis cinerea* by triggering JA- and ET-dependent signaling pathways, but not SA-induced pathway

In order to regulate the complex interactions with the microorganisms, plants have developed inducible defense responses. The first line of defense, that is induced by the recognition of molecules, including the elicitors, is called plant innate immunity (Boller and Felix, 2009). Once the immunity is induced, the response is amplified by the induction of SA-, JA-, and ET-induced signaling pathways (Garcion et al., 2007; Dangl et al., 2013). These defense mechanisms work coordinately to regulate the plant-pathogen interactions, locally and systemically by priming the defense responses, including the systemic and induced acquired resistance (SAR and IAR) (Craig et al., 2009; Tsuda and Somssich, 2015). Here we proposed the possibility that HoFs might work as elicitor to induce a systemic protection against *Botrytis cinerea* (Figure 5). JA- and ET-related genes are induced after HoFs application (Figure 6, Table 1), but the SA-induced gene *PR1* is actually repressed (Supplementary Table 1). For decades, SA has been proposed to govern the induction of SAR, however, multiple reports have revealed that systemic defense responses are not regulated and induced only by SA but by an intricate and complex network that involves other phytohormones including JA and ET (reviewed by Conrath et al., 2015; Klessig et al., 2018). With this in mind, characterization of HoFs-induced defense responses warrants further studies.

### Exploring the pathosystem *Arabidopsis thaliana-botrytis cinerea* to characterize HoFs-induced defense mechanisms

Elicitors have the potential to be used in agriculture as an alternative to chemical fungicides, however, in order to optimize their application and activity on the field, it is necessary to know and characterize their mode of action (Wiesel et al., 2014). In this report, we used the well characterized pathosystem *Botrytis cinerea*-*Arabidopsis thaliana* to identify the transcriptomic changes induced by HoFs (Figure 6, Table 1, Supplementary Table 1). Using genetic, molecular and *omics* tools applied on different plant models, including *Arabidopsis thaliana*, plant-microbe and microbe-microbe interactions, have been characterized at the molecular level (Kroll et al., 2017). In the plants, this characterization includes, the analysis of the early events during the beneficial and pathogenic interactions (Zipfel and Oldroyd, 2017), the transcriptional regulation of plant defense responses (Birkenbihl et al., 2017) and the elicitor-mediated activation of plant immunity (Cheng et al., 2018). On the other hand, the molecular analysis of the pathogens *Corynespora cassiicola* and *Botrytis cinerea* also has also been improved with the identification of the genomic sequence and the transcriptomic characterization during the interaction with the plants (Windram et al., 2012; Shrestha et al., 2017; Van Kan et al., 2017). Now, with all this available information and with the HoFs-induced DEGs identified from our work, further studies are warranted, that might help us to understand the molecular defense mechanisms induced by HoFs.

### Triggered transcriptional modulation of plant defense responses is broadly BCAs-specific

Only two BCAs have been characterized by analyzing genome-wide transcriptional changes in *Arabidopsis thaliana*, using the bacterium *Ralstonia solanacearum* ΔhrpB mutant strain and the fungus *Trichoderma harzianum* (Feng et al., 2012; Morán-Diez et al., 2012). In order to identify similarities between the transcriptome induced by different-origin BCAs, we compared the DEGs from these two reports and those induced by yeast-derived HoFs (Figure 7). Remarkably, we observed that only 9 DEGs are shared in response to *Trichoderma harzianum* and *Ralstonia solanacearum* treatments and that there were no similarities with HoFs treatment (Figure 7). In agreement with these observations, it was previously reported that the expression of JA-related genes was down-regulated after *Ralstonia solanacearum* induction (Morán-Diez et al., 2012), while we determined that after HoFs treatment these genes were up-regulated (Figure 6B). Similar differential responses have been described in others plant-microbe interactions, for example, the pathogenic bacterium *Pseudomonas syringae* has been shown to induced the SA-induced signaling pathway (Grant and Jones, 2009; Verhage et al., 2010), while the fungus *Botrytis cinerea* induced JA- and ET-signaling pathways (Thomma et al., 2001; Glazebrook, 2005). To further highlight the complexity of these interactions, other reports have also shown contradictory results on the phytohormone-dependent responses induced by biotrophic and necrotrophic pathogens, since complex cross-talks and multifactorial dependence between SA-, JA-, and ET-signaling pathways have been described (Koornneef and Pieterse, 2008; Pieterse et al., 2009; Hael-Conrad et al., 2015). These observations suggest that both, the triggered defense mechanisms and the protective effect against a particular pathogen(s) are differentially regulated depending of the origin of BCAs.

Summarizing, HoFs induce local and systemic defense responses to broad host-range necrotrophic fungi. HoFs induce a transcriptional reprograming of *Arabidopsis thaliana* plants, and this genome-wide information can be used as starting point to understand the molecular basis of HoFs-triggered responses. Future work is now directed to characterize the biochemical nature of HoFs, including the chemical identity/identities of the elicitor(s).

## Author contributions

DF, CS, AT, KS-E and MS conceived and designed the experiments. MF-S, MT, WA, EP, and DF performed the experiments. DF, CS, AT, and MS wrote and revised the paper. All authors approved the final version of the manuscript.

### Conflict of interest statement

The authors declare that the research was conducted in the absence of any commercial or financial relationships that could be construed as a potential conflict of interest.

## Abbreviations

HoFs: *Hanseniaspora opuntiae*-Filtrates
hpt: hours post treatment
hpi: hours post inoculation
PDA: potato dextrose agar media
YNB: yeast nitrogen base media.
